# Supplementary material for: Recruitment and retention interventions in surgical and wound care trials: A systematic review
Source: PLoS One. 2023 Jul 20;18(7):e0288028. doi: 10.1371/journal.pone.0288028 (PMC10358880; doi:10.1371/journal.pone.0288028)
Supplement: S1 File — (DOCX) [file pone.0288028.s001.docx]

**Supplementary Document 1: Full Search Strategy**

MEDLINE

1 Patient Selection/

2 ((participat$ or recruit$ or enrol$) adj4 trial?).tw.

3 Informed Consent/

4 informed consent.tw.

5 recruitment.ab. /freq=2

6 participation.ab. /freq=2

7 minimi$ or prevent$ or lessen$ or decreas$ or reduc$

8 #7 adj2 attrition

9 #7 adj2 drop$-out$

10 #7 adj2 dropout$

11 #7 adj 2 withdrawal$

12 (loss adj2 follow-up).ab,ti

13 (loss adj2 followup).ab,ti

14 Attrition or drop-out or dropout or followup or follow-up

15 (strategy$ adj2 #14

16 increas$ or encourage$ or maximi$ or promot$ or improve$

17 #16 adj2 retention

18 strateg$ adj2 responses

19 #16 adj2 responses

20 questionnar$ adj 3 respons$

21 strateg$ adj2 (questionnaire$ adj3 response$))

23 (retention adj2 strateg$)

24 Retention rate$

25 Retention adj2 method$

26 Retention ajd2 technique$

27 Attrition rate$

28 (questionnaire$ adj3 (response$ adj2 method$)).ab,ti

29 (questionnaire$ adj3 (response adj2 technique$)).ab,ti

30 (questionnaire adj response rate$).ab,ti.

31 (difficult$ adj2 (retain$ or retention)).ab,ti.

32 Patient Dropouts/

33 #1 and #2 …. And #32

#33 randomized controlled trial.pt.

#34 controlled clinical trial.pt.

#35 randomized.ab.

#36 placebo.ab.

#37 clinical trials as topic.sh.

#38 randomly.ab.

#39 trial.ti.

#40 #33 or …or#39

#41 comment.pt.

#42 editorial.pt

#43 #40 not (#41 or #42)

#44 exp animals/ not humans. sh.

#45 #43 not 44

#46 #33 and #45

47 Surgical procedures

48 #46 and #47

49 Wound$ (wounds and injuries) (also research-related injuries, injuries, wounds?)

50 #46 and #49

EMBASE

1 ((participat$ or recruit$ or enrol$ or enter$ or entry) and (trial? or study)).ti

2 (select$ adj3 (participants or patients or controls)).tw

3 recruit$.ab. /freq=2

4 participat$.ab. /freq=2

5 research.tw

6 2 and (3 or 4 or 5)

7 (informed consent or consent process$ or consent procedure?).tw

8 1 or 6 or 7

9) minimi$ or prevent$ or lessen$ or decreas$ or reduc$).ab,ti

10 (#9 adj2 attrition).ab,ti

11 (#9 adj2 drop-out).ab,ti

12 (#9 adj2 drop-out$).ab,ti

13 (#9 adj2 drop$-out).ab,ti

14 (#9 adj2 dropout$).ab,ti

15 (strateg$ adj2 drop$-out).ab,ti

16 (strateg$ adj2 dropout$).ab,ti

17 (loss adj2 follow-up).ab,ti

18 (lost adj2 follow-up).ab,ti

19 (loss adj2 followup).ab,ti

20 (lost adj2 followup).ab,ti

21 (#9 adj2 withdrawal).ab,ti.

22 (#9 adj2 withdrawal$).ab,ti

23 (strateg$ adj2 attrition).ab,ti

24 (strateg$ adj2 drop-out).ab,ti

25 (strateg$ adj2 dropout).ab,ti

26 (strateg$ adj2 follow-up).ab,ti

27 (strateg$ adj2 followup).ab,ti

28 (increas$ or encourage$ or maximi$ or promot$ or improve$).ab,ti

29 (encourag$ adj2 retention).ab,ti

30 (#28 adj2 retention).ab,ti

31 (strateg$ adj2 response$).ab,ti

32 (strateg$ adj2 (questionnaire$ adj3 response$)).ab,ti

33 (#28 adj2 (questionnaire$ adj3 response$)).ab,ti.

34 (#28 adj2 response$).ab,ti

35 (retention adj2 strateg$).ab,ti

36 retention rate$.ab,ti

37 (retention adj2 method$).ab,ti

38 (retention adj2 technique$).ab,ti

39 attrition rate$.ab,ti

40 (questionnaire$ adj3 (response$ adj2 method$)).ab,ti

41 (questionnaire$ adj3 (response adj2 technique$)).ab,ti

42 (questionnaire adj response rate$).ab,ti

43 (difficult$ adj2 (retain$ or retention)).ab,ti.

44 Participant Dropouts/

45 #9 or #10 or #11 or 12 or 13 or 14 or 15 or 16 or 17 or 18 or 19 or 20 or 21 or 22 or 23 or 24 or 25 or 26 or 27 or 28 or 29 or 30 or 31 or 32 or 33 or 34 or 35 or 36 or 37 or 38 or 39 or 40 or 41 or 42 or 43 or 44

46 #8 or ##45

47 random$.tw

48 placebo$.ti,ab,sh

49 double-blind$.tw

50 #47 or #48 or #49

51 #46 and #50

52 surgery/

53 #51 and #52

54 Wound$ (all) or Wound/

50 #46 and #49

Cochrane Library

*Recruitment*

1 Patient selection

2 Recruitment

*Retention*

1 Patient Drop out

2 Questionnaire

3 Response

4 Retention

ORRCA database

*Recruitment Domains*: Trials design; pre-trial planning; trial conduct; recruitment information needs; recruiter differences; incentives; other

Retention Domains: Data collection; participants; sites and site staff; central study management; study design

*Host Design*: RCT; Factorial RCT; Cross over RCT; Cluster RCT

*Health Intervention Type*: Surgery

*Health Intervention Type*: Medical Devices

Northern Ireland Hub for Trials Methodology Research SWAT Repository Store

1. Surgical

2. Surgery

3. Wound
