# Supplementary material for: Recruitment and retention interventions in surgical and wound care trials: A systematic review
Source: PLoS One. 2023 Jul 20;18(7):e0288028. doi: 10.1371/journal.pone.0288028 (PMC10358880; doi:10.1371/journal.pone.0288028)
Supplement: S2 File — (DOCX) [file pone.0288028.s002.docx]

**Supplementary Document 2: GRADE Assessment of Included Studies**

| **Study ID** | **Certainty of Evidence (GRADE)** |
| --- | --- |
| Abd Elsayed et al (2012) | ++OO |
| Brubaker et al (2019) | ++OO |
| Watson et al (2017) | +OOO |
| Mitchell et al (2020a) | ++OO |
| Mitchell et al (2020b) | ++OO |
| Sarathay et al (2020) | ++OO |
| Eccles et al (2002) | +++O |
| Renfroe et al (2002) | ++OO |
| Donovan et al (2003) | +OOO |
| Jefferson et al (2018) | +++O |
| Parker et al (2022) | ++OO |
| Agni et al (2022) | ++OO |
| Coleman (2022) | +++O |
