# Supplementary material for: Recruitment and retention interventions in surgical and wound care trials: A systematic review
Source: PLoS One. 2023 Jul 20;18(7):e0288028. doi: 10.1371/journal.pone.0288028 (PMC10358880; doi:10.1371/journal.pone.0288028)
Supplement: Attachment — Submitted filename: Response to Reviewers 03.02.2022_.docx [file pone.0288028.s003.docx]

| **Reviewer 1** | |
| --- | --- |
| The introduction introduces appropriately the reader to the topic. However, it doesn't appear clear why the authors decided to investigate both surgical trials and wound trials. Please argument why the two populations can be analysed together. | We are pleased that the introduction provided a helpful summary of the topic but apologise it was not clear why the populations were combined. We have updated the introduction section to reflect that surgery often leads to wounds and therefore the populations are likely to be similar, hence the grouping here. |
| Discussion "findings were not unsurprising; additional visits to sites and recruitment by a urologist rather than a nurse were more expensive. Retention at subsequent time points was not assessed in any of the included retention SWATs."  In this paragraph I would expect the authors to justify why they find the results not unsurprising. Please, consider also revise the terms "not unsurprising”, which may be difficult to understand for someone not speaking in English as first language. | We apologise that this wording was ambiguous. The Discussion has been updated accordingly to reflect the results were expected given the resource implications associated with the expensive interventions. |
| Moreover, it would be nice to comment the replicability of the results in the discussion in relation to the limited part of the population described (white, males). | This suggestion to update the discussion to reflect the EDI of participants has been actioned accordingly to reflect the population is predominantly older patients, largely white ethnicity. |
| **Reviewer 2** | |
| For both the abstract and the introduction I think the rationale for the need for this review could be clearer. As written it states that recruitment and retention are an ongoing issue for trials. There are lots of operations and people living with chronic wounds and therefore assessment of this context is important. I would argue there needs to be a more direct link between recruitment and retention and surgical and wound care trials, rather than high numbers in clinical care. For example, do they routinely under-recruit/retain – what is the problem that this review is addressing? This needs strengthened. | We are sorry that the rationale for the review was not clear. We have updated the abstract and introduction to make clear the difficulties with recruitment/retention in surgical trials and given that surgery often leads to wounds and therefore the populations are likely to be similar, hence why these were grouped together. |
| Within abstract methods section helpful to state ‘within a surgical or wound based host randomised controlled trial’ so as to be consistent with terminology throughout re host trials – which will help readers. | We thank the reviewer for this note re consistency and have updated the Abstract – Methods accordingly. |
| There isn’t any information on the analysis methods used in the methods section. | We thank the reviewer for noting this missingness. The Abstract – Methods has been updated to reflect the analysis methods used. |
| Results section states 11 studies were included but in the main text it states 12 and 12 studies are included in the tables. | We apologise for the inaccuracy of the reporting here. This was clearly missed when we updated the manuscript with an updated search. The Abstract – Results section has been updated accordingly. |
| Within the opening sentences there are several uses of ‘their’. Sometimes it isn’t clear which ‘their’ the author is referring to. Maybe clearer to be more specific e.g sentence 2 – The validity and reliability of RCTs is highly dependent on recruiting and retaining….’ | We appreciate that the use of their may have caused ambiguity in the initial sentences of the Introduction and have amended this accordingly for clarity. |
| Within introduction sometimes the ‘interventions’ are referred to as methods sometimes strategies – would be helpful to be consistent. | We thank the reviewer for their suggestion here – the manuscript has been reviewed and updated throughout for consistency |
| Point re rationale made above for abstract also needs addressed here. | As above we have updated the abstract and introduction to better reflect the rationale. |
| I didn’t understand the second section of the last sentence of the second paragraph. | We apologise this point was not clear. On review this duplicates information provided in Paragraph 3 and so has been removed |
| Last sentence of paragraph 3. The point being made re evidence from quasi or non-randomised designs – is this in relation to the design of the recruitment and retention trial or the host trial? I didn’t then understand how this links to the point re ‘real’ trials. Isnt that more that some trials included in the Cochrane recruitment review were hypothetical? | We have made clearer that the quasi and non randomised designs are relating to the SWAT and have also made reference to hypothetical designs as suggested. We have removed the reference to ‘real’ trials for avoidance of confusion |
| Sentence that states studies were not eligible if they were hypothetical needs clarification. If the host parent trials was hypothetical? The SWAT? Both? | The Methods – Eligibility Criteria section has been clarified as requested. |
| Were any other types of studies excluded? | The inclusion criteria note studies as eligible for inclusion if the host trial and SWAT were randomised. We have amended the exclusion criteria to make clear non randomised or quasi randomised designs were not eligible to make this clearer. |
| Would be helpful to state how the outcomes of recruitment and  retention were defined. | As requested definitions of recruitment and retention have been added to the Methods – Outcome data section |
| Was unit of randomisation collected as a SWAT characteristic? | We did not separately extract unit of randomisation but this was collected as part of the number of participants recruited to each arm, whereby this was noted as the number of sites for relevant studies. We have updated the Methods – Data Items to include this. |
| In Synthesis section it states a flowchart ‘will be presented’ – needs changed to ‘are’ | We apologise for the oversight in tense here. This has been updated accordingly. |
| Reports that 12 studies were included – ensure address mismatch with abstract | We apologise for the inaccuracy of the reporting here. This was clearly missed when we updated the manuscript with an updated search. The Abstract – Results section has been updated accordingly. |
| When stating studies use a direct approach – can you give an i.e. to hep the reader. | This is a useful addition and we have added as suggested to the Results – Study Characteristics section. |
| Likewise when stating postal follow up – maybe include postal questionnaire follow up. | This is a useful addition and we have added as suggested to the Results – Study Characteristics section. |
| The results state that one intervention (patient information video decision aid) was evaluated in ‘multiple’ studies. It was evaluated in 2 studies – why weren’t these studies considered for meta-analysis? | The authors have re-reviewed this decision and agree that the two interventions were too heterogeneous to warrant combining via meta analysis. We have updated the results section to remove any ambiguity here. |
| Risk of bias – states 11 studies were included | We apologise for the inaccuracy of the reporting here. This was clearly missed when we updated the manuscript with an updated search. The Results – Risk of Bias section has been updated accordingly. In undertaking this we noted that the assessment for Parker et al was inadvertently missed from the reporting – this has been added accordingly. |
| Recruitment section o second para opens stating 4 of the 6 SWATs – but weren’t there 7? 4 on consent info, 1 on study set up and 2 on training.  o I think it would be helpful to provide more information on ‘consent information’ interventions. This could mean the content and/or the mode pf delivery of information has been modified. Would be helpful to provide further details – could consider using similar categories to the Cochrane review.  o Para 7 – should be ‘were’ not ‘was’ identified. | We apologise for the inaccuracy of the reporting here. This was clearly missed when we updated the manuscript with an updated search. The Results – Recruitment section has been updated accordingly.  This is a helpful suggestion and we have updated the manuscript to make clear the types of consent information interventions.  We apologise for the oversight in tense here. This has been updated accordingly. |
| Retention section o Last sentence of first para – were these host trials that were factorial?  o 2nd para last sentence – I wasn’t clear why the authors report the SWATs focused on provision of consent information and study set up – isn’t that relevant for the recruitment interventions not retention. | It was the SWATs which were factorial – we have updated the Results – Retention section for clarity  We thank the reviewer for spotting this oversight- you are correct the provision of consent information and set up should not be detailed here. We have revised this section to report the retention interventions correctly. |
| Could the authors comment on why the following paper wasn’t included as some of the included trials are surgical–  o Coleman E, Arundel C, Clark L, Doherty L, Gillies K, Hewitt C et al. Bah humbug! Association between sending Christmas cards to trial participants and trial retention: randomised study within a trial conducted simultaneously across eight host trials BMJ 2021; 375 :e067742 | At the time of initial search this publication was not available so not included and it appears the subsequent search did not pick up this publication either, possibly due to the timing of indexing. We acknowledge that this should have been included however and so have added this in accordingly. |
| Discussion needs edited based on ‘consent information; interventions description being expanded | We have updated the Discussion section to reflect the types of consent information interventions included. |
| 2nd para last sentence – reads that replications are required due to heterogeneity of interventions. But replications are also required due to small sample sizes which means that individual studies don’t provide high quality, reliable, evidence. This point needs to be covered in discussion | This is an excellent point – we have updated this section of the Discussion to reflect the impacts of the sample sizes here. |
| Would be helpful to also cover priorities for recruitment and retention intervention testing as stated in Cochrane reviews. | This is a helpful suggestion and one which has now been reflected on within the Discussion section |
