# Supplementary material for: Recruitment and retention interventions in surgical and wound care trials: A systematic review
Source: PLoS One. 2023 Jul 20;18(7):e0288028. doi: 10.1371/journal.pone.0288028 (PMC10358880; doi:10.1371/journal.pone.0288028)
Supplement: Attachment — Submitted filename: Response to Reviewers 12.05.2023.docx [file pone.0288028.s004.docx]

| **Reviewer 1** | |
| --- | --- |
| Abstract: the introduction is not clear and it doesn't introduce properly the two populations under scrutiny and why retention is a problem. | We apologise that the introduction was unclear as to the two populations under scrutiny. We have sought to revise the introduction section to make this clearer whilst remaining within the confines of the journal word count for the abstract. |
| In the abstract the authors state there are 7 articles included for recruitment, but then they say "four out of the SIX". | We apologise for the oversight here when revising this manuscript. This has been corrected accordingly. |
| Introduction: as the previous reviewer undelined, the authors didn't justify clearly why surgical and wound care trials against all other trials, need to be addressed in terms of retention strategies, there needs to be a more direct link between recruitment and retention and surgical and wound care trials, rather than high numbers in clinical care.  Moreover, in the comments to the authors, the authors declare that they included 12 articles, but in reality in the manuscript there are 13 articles. | We are sorry that the amendments made to the introduction did not satisfactorily deal with the previous reviewer comments. We have made further revisions to the introduction to note the difficulties in both recruitment and retention in these trial types.  We have thoroughly checked the manuscript to ensure that it is clear that 13 articles in total were included in the review. |
| Analysis: if there are 13 RCTs, it should be justified why the authors talk about 14 interventions. | We apologise for the inaccuracy here. The analysis section has been amended to remove details of the number of interventions for avoidance of any confusion. |
| A further record was subsequently identified for inclusion given 62.5% of included studies were surgical or wound care related". This sentence is not clear to me: Why this further record has not been included among the 13? why is it relevant to state that 62.5% of the studies where surgical or wound care related? | We apologise for the oversight here when revising the manuscript. The original number of studies included should have been reported as 12, with the additional record increasing this to 13. This has been updated accordingly for clarity.  We suggest it is relevant to report the proportion of surgical/wound care studies in the final included record, given this is a simultaneous SWAT (i.e., conducted in multiple studies at the same time) and not all the studies included would meet the inclusion criteria for this review. |
| Page 24: please consider changing "results where not unsurprising". | We thank the reviewer for this comment. We have re reviewed the manuscript and amended this sentence in relation to the cost effectiveness of recruitment strategies. |
| Discussion: in the last paragraph the authors state that "Half of the studies included used clinic follow-up either alone or in conjunction with remote follow-up. It would be interesting to discuss what are the forms of remote follow up.  A recent systematic review by Mette Brøgger-Mikkelsen et al (2020) hightlighted that Online recruitment was both superior in regard to time efficiency and cost-effectiveness compared with offline recruitment and this could be an effective strategy to improve the low attendance at face-to-face visits in trials.  Moreover, the systematic review cited by the authors in the last paragraph needs to be correctly cited. | We have added clarification to this sentence to reflect that the remote follow up methods reported were postal. We thank the reviewer for flagging the review by Mette Brogger Mikkelsen et al but have not commented further on this given neither online recruitment nor follow up methods were used by any of the included trials.  We apologise for the incorrect placing of this citation. This has been corrected accordingly. |
